# Canada 150

**Published:** 2017-12-15

**Authors:** Marcel D’Eon

**Affiliations:** University of Saskatchewan

As we put 2017 behind us, I am dedicating this editorial to the celebration of Canada’s 150^th^ birthday. I was fortunate to be in Ottawa for the month of August where I took in several Canada 150 events and displays, the most memorable and inspiring being MosaïCanada in Gatineau (created by the world famous Mosaïcultures internationales de Montréal http://english.mosaicanada.ca/). So impressed and moved was I by the beauty of the sculptures, the imagination of the designers, and the stories that were told* that I toured the display twice on my first visit and then again when I was able to make a second pilgrimage three weeks later. I am pictured here with one of my three sisters in front of a tranquil and serene Mother Nature. Of course I am wearing my Canada T-shirt! Happy 150^th^, Canada!

The CMEJ is a full eight years old now. Staring out with two issues a year, we grew to three issues in 2016 and are now closing out 2017 with our fourth issue! Coincidentally, this issue includes authors from six different medical schools in Canada and Associate Editors from an additional five different medical schools. This was very much a cross-country collaboration, a coast-to-coast partnership (with 11 of the 17 Canadian medical schools contributing) that continues to make the CMEJ a national treasure.

**Figure:**
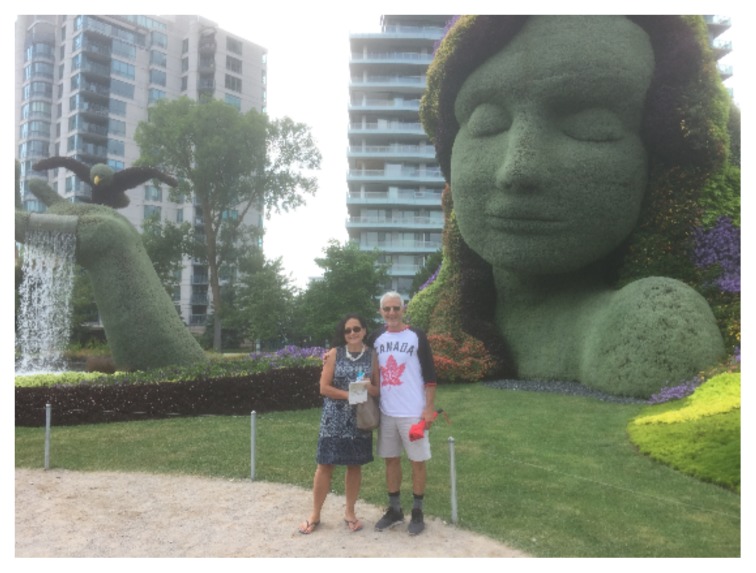


In our lead article, “Can an educational based intervention by a pharmacist improve the quality of prescription?” Carceller-Blanchard and her team from the University of Montreal measured the effectiveness of an additional two-hour lecture to junior pediatric residents by a pharmacist on rates of prescription errors and quality of prescription. There were 11 residents who had the lecture and 15 in a control group. The researchers’ included the first 50 prescriptions made by each resident. While this study informs us on the most frequent types of errors and prescription quality issues and while the data are promising, the authors conclude that the two-hour lecture was insufficient to reduce prescription errors among junior pediatric residents. I wish to point out to the readers that publishing negative studies is helpful for both researchers and practitioners. It is important to be able to identify reasons why our hypotheses were not correct – likely our choices of learning strategy or execution or both. Perhaps our methods were not up to the task. Unfortunately, such candor and humility is not always on display. In a paper published some years ago in a different journal, those authors reported a study where an extensive curricular innovation based on adult learning theory was poorly designed and implemented but they blamed the students’ lack of maturity for the lack of success.^1^

Barinder Singh and his team from Queen’s University, Kingston, wrote “Canadian residents’ perceptions of cross-cultural care training in graduate medical school.” They emailed the Cross Cultural Care Survey^2^ to 450 residents and then compared psychiatry, family medicine, and other program residents’ reports of training, preparedness, and skillfulness in delivering cross-cultural care. Seventy-three (16%) residents responded. The authors found that residents in psychiatry and family medicine reported significantly more training for and formal assessment of cross-cultural care than residents in other programs. Puzzlingly (and alarmingly), there were no significant differences in preparedness and skillfulness. How is it, then, that residents who did not receive much training in this challenging area of communication still believed that they were about as skilled as those who had benefited from direct instruction and assessment? Perhaps they felt that such skills were really just intrinsic to being a good doctor and that most everyone (including them) was fairly good at this skill. Maybe they did not see what they did not know to look for. I have noticed that in our medical schools while we have PhD trained experts in microbiology and physiology and renowned specialists in diabetes and laparoscopic surgery (for example), rare is it that a medical school employs full time experts with graduate training in communication or history of medicine or psychology? Clearly we can’t teach what we don’t know we are missing.

Elena Scali and co-authors from the University of British Columbia (UBC) have given us “Senior medical students’ awareness of radiation risks from common diagnostic imaging examinations.” Senior medical students represent future physicians who commonly refer patients for diagnostic imaging studies that may involve ionizing radiation. The purpose of this study was to investigate students’ awareness of radiation exposures and risks. An anonymous multiple-choice cross-sectional questionnaire was distributed to final year medical students at UBC to assess knowledge of radiation-related risks from exposure to common diagnostic examinations. While the majority stated that knowledge of radiation doses of common imaging examinations was important, only 12% of them routinely discussed radiation-related risks with patients, only 24% correctly identified gonads as the most radiation-sensitive tissue, and 54% underestimated the risk of a fatal cancer from an abdominal CT in an adult. It is indeed important for medical students to know the risks and to be able to discuss these with patients. How important? Likely more important than 15–55% of other not so important material that still lies encrusted on the interior walls of medical school curricula!^3^

Megan Delisle and her team from the University of Manitoba wrote the “National survey of mentorship in Canadian general surgery residency programs: Where are we and what do we need?” The benefits of mentorship on residents are well established so they set out to obtain general surgery residents’ and program directors’ perspectives on resident mentorship. They developed an electronic survey distributed to all 601 general surgery residents in Canada. All 17 program directors were invited for telephone interviews. Ninety-seven percent of responding residents felt mentorship was important but only 67% identified a mentor and only 53% reported a mentorship program. Most residents who identified a mentor were satisfied with the mentorship they received. Overall, residents strongly favoured having a mandatory mentorship program. Of the eleven program directors who were interviewed, most were satisfied with current resident mentorship but acknowledged that improvements could be made. The authors concluded that general surgery programs in Canada should provide mentorship opportunities and address local barriers. Of course, but how?

Alexander Kiss and his team from Switzerland and Germany aimed to determine whether (1) GPs who teach students clinical skills could also provide feedback for student reflective writing, and (2) an instruction letter for these GPs specific to providing feedback on reflective writing increased students’ satisfaction. GPs were randomized to the two study arms using block randomization. The intervention group of GPs received specific instructions on providing feedback to students’ reflective writing. Students completed satisfaction questionnaires on feedback received on clinical skills and reflective writing. Eighty-three physicians participated: 38 were randomized to the control, 45 to the intervention group. As reported in their article, “Students’ satisfaction with general practitioners’ (GP) feedback to their reflective writing: a randomized controlled trial,” they found that students were very satisfied with the feedback they received regardless of having had instructions on providing feedback or not. GPs who gave students feedback on clinical skills also provided highly rated feedback on reflective writing. This is good news since we know that reflective writing is increasingly being used in medical education and that feedback to students is essential while resources are scarce.

Karen Ethans’ multi-centre team (University of Manitoba; UBC; Island Health, Nanaimo, BC) submitted “The virtual hallway consult as an effective means of continuing professional development in physiatry.” Many complex cases in rehabilitation medicine have no readily available answers so physicians turn to other physician colleagues for advice. The Virtual Spinal Cord Injury Hallway was created to provide a simple tool to extend hallway consultations to colleagues across the country. On this invite-only Yahoo Groups site, members post questions, all members receive the posts by email, and any member may respond. For over 13 years The Virtual Spinal Cord Injury Hallway has been running successfully (with 38 members and 2124 messages within over 300 conversations). The Virtual Spinal Cord Injury Hallway is a very successful, secure, invite-only site requiring low-maintenance and no funding. In fact, it is likely more than the complexity of the cases that drives physicians to ask other physicians for advice. We can make the case that just like teaching and other human endeavours, medicine is a social practice.^4^

Linzi Williamson and a team from the University of Saskatchewan and the Saskatchewan Prevention Institute wrote “A needs assessment on addressing environmental health issues within reproductive health service provision: Considerations for continuing education and support.” One hundred and thirty-five nurses and physicians working in Saskatchewan completed a survey designed to explore their knowledge of the impact of environmental toxicants on maternal and infant health and to describe current practices and needs related to education. Although participants considered it important to address environmental health issues (EHI) with patients, in actual practice they do so with only moderate frequency. Participants reported low levels of knowledge about EHIs’ impact on health, and low levels of confidence discussing them with patients. This is a similar even companion study to Elena Scali and co-authors’ paper “Senior medical students’ awareness of radiation risks from common diagnostic imaging examinations.” One would be hard pressed to explain how some topics currently privileged in our medical schools are more important than addressing these clinically relevant patient risks.

In their piece “Perceptions, practice, and ownership: Interpersonal continuity experiences in a family medicine residency,” Ann Lee and her team from the University of Alberta carefully extracted data from electronic medical records and used the Usual Provider Continuity (UPC) Index to calculate continuity of care rates. They also conducted semi-structured interviews using constant comparative analysis to identify emerging themes related to continuity of care experiences. They learned that residents had low UPC rates and preceptors had higher rates. There were variable experiences with interpersonal continuity not apparent from UPC rates; both preceptors and residents expressed the belief that lack of “ownership” of patients (a feeling or situation of special responsibility) was a significant barrier to interpersonal continuity. The authors recognize that the term “ownership” which was used commonly by participating residents and physicians is somewhat problematic, both its denotation and connotations. Perhaps this is worth a conversation or more.

MacPherson and Emberley from Queen’s University and Memorial University of Newfoundland wrote “Ethics learning needs of pediatric residents: An interprofessional needs assessment.” Ethics education is a required but somewhat neglected component of pediatric residency training. The authors identified pediatric residents’ ethics learning needs using a multisource (360 degree) tool to rate the importance of twelve ethics themes. One-way ANOVA was used to determine differences between the groups, followed by post-hoc testing. Pediatricians and the other health care professionals did not rate any ethics themes higher than residents. High priority ethics topics were identified, especially by residents, implying the need for curriculum revisions. And I can say the same thing about the curricular implications of this research as I did for the Scali and Williamson studies: some content is more important than others and far too often the biomedical is prioritized over the psychosocial. It happens so often and is so pervasive that most of us don’t even notice this “elephant” in our medical school curricula.^5^

In “The physician as person framework: How human nature impacts empathy, depression, burnout, and the practice of medicine,” Lester Liao reminds us that “physicians are first and foremost people.” He then describes five features or characteristics that make us human with implications for physicians. Physicians and patients share a common humanity; people integrate and in fact don’t really have separate personal and professional lives; they are dynamic, thoughtful, and emotional; they are finite (yes, even physicians); and they are moral beings. He also contends that this framework applies equally to medical students and residents.

Liao’s framework is amplified and enhanced by Damon Dagnone’s life experiences, forged through challenging personal and professional circumstances. You can read more about his family’s tragic and profound journey in the explanation of the footprint appreciatively used as a cover image to this issue of the CMEJ (discovered at White Coat Warm (he)Art at the 2017 Canadian Conference on Medical Education in Winnipeg, MB, https://www.teachingmedicine.com/galleries/View.aspx?gallery=22). Unapologetically, I strongly encourage everyone to read his brief account of their experiences.

Lori Hanson, from the University of Saskatchewan, reviewed the book *Revitalizing health for all: Case studies of the struggle for comprehensive primary health care* edited by Ronald Labonte, David Saunders, Corinne Packer and Nikki Schaay.^6^ Hanson notes that 2018 marks the fortieth anniversary of the Alma Ata Declaration on Primary Health Care. This is a good reason to release *Revitalizing Health for All: Case studies of the struggle for primary health care,* a compilation of experiences and research into the ideal of “Health for all through Comprehensive Primary Health Care” (CPHC). Hanson believes that overall the book presents a compelling though problematic picture. Well designed and implemented research on CPHC remains rare. While struggling to find resources for even small projects adhering to CPHC principles, decision and policy makers are reluctant to allocate resources to research, measurement and/or meaning-making. While she criticizes the book for not addressing lessons learned about *methodological* issues encountered in this project, Hanson enthusiastically recommends it.

With this issue we are closing out the eighth year of the CMEJ in style with author and editor representation from across the country and over the ocean. We hope that the articles and images of Volume 8(4) are as inspiring as many of the Canada 150 displays and will be as prolific at generating new programs and productive research as Mother Nature herself!

## Footnotes

* One of the many magnificent MosaïCanada displays captured the celebration after Henderson scored the game winning goal to defeat the Russians in 1972. I remember exactly where I was that day: in class with everyone including the professor huddled around a small TV. (Please note: I did not write that Canada defeated the “Russian hockey team” because it was not a game and certainly not just about hockey.)
